# The use of honey bees (*Apis mellifera* L.) to monitor airborne particulate matter and assess health effects on pollinators

**DOI:** 10.1007/s11356-024-33170-8

**Published:** 2024-04-13

**Authors:** Giulia Papa, Marco Pellecchia, Giancarlo Capitani, Ilaria Negri

**Affiliations:** 1 Dipartimento Di Scienze Delle Produzioni Vegetali Sostenibili (DIPROVES), Università Cattolica del Sacro Cuore, Via Emilia Parmense 84, 29122 Piacenza, Italy; 2 KOINE’–Consulenze Ambientali, Montechiarugolo, Italy; 3 Dipartimento di Scienze dell’Ambiente e della Terra (DISAT), Università Milano Bicocca, Milano, Italy

**Keywords:** Bee, Bioindicator, PM, Human health, Environmental impact, One-health approach

## Abstract

The honey bee *Apis mellifera* has long been recognized as an ideal bioindicator for environmental pollution. These insects are exposed to pollutants during their foraging activities, making them effective samplers of environmental contaminants, including heavy metals, pesticides, radionuclides, and volatile organic compounds. Recently, it has been demonstrated that honey bees can be a valuable tool for monitoring and studying airborne PM pollution, a complex mixture of particles suspended in the air, known to have detrimental effects on human health. Airborne particles attached to the bees can be characterised for their morphology, size, and chemical composition using a scanning electron microscopy coupled with X-ray spectroscopy, thus providing key information on the emission sources of the particles, their environmental fate, and the potential to elicit inflammatory injury, oxidative damage, and other health effects in living organisms. Here, we present a comprehensive summary of the studies involving the use of honey bees to monitor airborne PM, including the limits of this approach and possible perspectives. The use of honey bees as a model organism for ecotoxicological studies involving pollutant PM is also presented and discussed, further highlighting the role of the bees as a cornerstone of human, animal, and environmental health, according to the principles of the “One Health” approach.

## Introduction

The World Health Organization describes the particulate matter (PM) as an air pollutant that consists of a heterogeneous mixture of soil and liquid particles suspended in the air (World Health Organization 2013). The particulate pollution may be of natural or anthropogenic origin. Natural particles include sea salts, volcanic ash, wind-blown dust, soil particles, fungal spores, pollen, forest fire ashes, and the oxidation products of biogenic volatile organic compounds (Kelly and Fussell 2012; Kim et al. 2015). Anthropogenic sources include fossil fuel combustion (e.g., vehicles and power plants), erosion of the pavement by road traffic, abrasion of brakes and tires, industrial emissions (e.g., from cement, metals, ceramic and bricks production), building, smelting, quarrying and mining activities, agricultural activities, cigarette smoking, and wood stove burning (Kelly and Fussell 2012; World Health Organization 2013; Kim et al. 2015).

Anthropogenic and natural sources can emit primary or secondary particles into the atmosphere (Juda-Rezler et al. 2011; Kelly and Fussell 2012). Secondary PM is generated in the atmosphere by photochemical gas-to-particle reactions, such as sulphur dioxide, nitrogen oxides, ammonia, and non-methane volatile organic compounds, that produce low-volatility substances which condense into solid or liquid phases (Harrison et al. 2000; Juda-Rezler et al. 2011; Kelly and Fussell 2012; World Health Organization 2013; Kim et al. 2015).

PM is classified according to its aerodynamic diameter (*d*_a_) as: coarse PM_10_ (*d*_a_ ≥ 2.5 µm and *d*_a_ < 10 µm), fine PM_2.5_ (*d*_a_ < 2.5 µm), and ultrafine PM_0.1_ (*d*_a_ < 100 nm) (Brook et al. 2010; Juda-Rezler et al. 2011; Kelly and Fussell 2012). PM between 0.1 and 1 μm in diameter can remain in the atmosphere for days or weeks and thus can be subject to long-range transboundary transport in the air (Juda-Rezler et al. 2011; World Health Organization 2013).

Chemical constituents in PM include minerals, metals, sulphates, nitrates, ammonium, and other inorganic ions, such as ions of sodium, potassium, calcium, magnesium and chloride, organic and elemental carbon, and polycyclic aromatic hydrocarbons (PAH) (Kelly and Fussell 2012; World Health Organization 2013; Kim et al. 2015; Harrison 2020). Moreover, biological components such as bacteria, viruses, fungi, mould, bacterial spores, and pollen may be found (World Health Organization 2013; Manisalidis et al. 2020).

Particulate matter is ranked as one of the leading causes of morbidity and mortality worldwide and with a significant impact on ecosystems’ health (World Health Organization 2013). While the scientific literature on atmospheric PM has increased enormously over the last decades, the role of the different chemical components of PM in inducing harmful effects is not yet fully known. Furthermore, the complex and highly variable composition of pollutant PM makes adequate monitoring and characterization extremely difficult. An alternative approach to assess PM pollution is biomonitoring and this paper aims to provide an updated framework on the use of the honey bee as an ideal tool for collecting spatial and temporal data on airborne PM at fine scales. An overview of the methodology based on the use of scanning electron microscopy coupled with X-ray spectroscopy to provide the chemical-physical characterization of airborne PM collected by the bees is also presented. Finally, a review of the most recent results in the ecotoxicology of bees exposed to particulate pollutants is provided, highlighting the role of the bees as a cornerstone of human, animal, and environmental health, according to the principles of the “One Health” approach.

## Impact of airborne PM on human and ecosystem health

Airborne particles may have a significant impact on human and ecosystem health. In humans, particles can penetrate within the respiratory system depending on their size (Kim et al. 2015; Manisalidis et al. 2020). In particular, PM_10_ can penetrate the respiratory tract below the larynx, while PM_2.5_ can penetrate deeply in the lung where the ultrafine fraction may cross the alveolar epithelium. Epidemiological studies suggest that airborne PM is responsible of several respiratory, cardiovascular and also gastrointestinal diseases following direct PM swallowing or ingestion of contaminated food (Wu et al. 2019; Kuo et al. 2022). Furthermore, neurological effects (e.g., Alzheimer’s disease, Parkinson’s disease, and neurodevelopmental disorders) have been observed in adults and children after long-term exposure to air pollutants (Maher et al. 2016; Manisalidis et al. 2020). Finally, dermatological studies suggest that airborne PM can also induce a number of skin pathological conditions (Diao et al. 2021).

According to toxicological studies, the shape of the particles may be critical for the interactions with biological systems, independently of their chemical composition. For example, fibrous materials, including silicate minerals (commonly referred as asbestos), ceramic, and vitreous fibers, may affect the defense mechanisms of the lungs (e.g., mucociliary clearance) and induces several diseases, such as cancer or mesothelioma. Also, fiber ingestion seems to be responsible for cancer in the gastrointestinal tract (Paris et al. 2017). Furthermore, cellular uptake is enhanced by the spherical morphology of the particles, which also tend to quickly circulate inside the blood vessel, while rod-shaped ones usually accumulate towards the vessel wall, where they may bind to wall receptors or cross the endothelium.

Epidemiological findings of PM health effects are supported by toxicological studies, which may involve in vitro (i.e., the use of cell cultures) or in vivo approaches (Reifferscheid and Buchinger 2017; Belden 2020), the latter including primarily vertebrate species (e.g., mice, rats, and fishes). The use of alternative in vivo models involving invertebrate species (e.g., nematodes, insects, and crustaceans) is increasing (Volta et al. 2020; Smoot et al. 2022; Papa et al. 2023). Among invertebrates, the use of bees in ecotoxicological studies are gaining much attention not only to assess risks to human health but also to the impact on ecosystems’ health and functioning. Ecotoxicological studies involving the bees clearly demonstrate that pollutants, including airborne PM, may affect the honey bee health even at sub-lethal doses. As a consequence, the ecosystem services provided by these insects are impaired, first of all pollination of both wild and cultivated plants (Papa et al. 2022).

## Airborne PM monitoring

The concentrations of PM associated with environmental hazard are regulated by European emission standards (Directive 2008/50/EC) which sets limits for PM_2.5_ and PM_10_ expressed as mass per unit volume of atmospheric air. In particular, according to the guidelines, PM_10_ and PM_2.5 _should not exceed 45 μg/m^3^ and 15 μg/m^3^ daily mean, respectively, or 15 μg/m^3^ and 5 μg/m^3^ annual mean, respectively. In current ground-based monitoring systems, PM_10_ and PM_2.5_ are collected using high volume samplers which draw a known volume of ambient air through a size selective inlet and a filter. The filters are weighed before and after sampling at an appropriate location (Gozzi et al. 2016).

Although this approach is useful in quantifying the presence of PM_10_ and PM_2.5_, it does not consider the fact that PM toxicity is mostly linked to the chemical-physical characteristics of the particles composing the mixture. Moreover, the filtering method features a complex post-processing and carries the risk of pore clogging. Also, the procedure does not provide information on the contribution of the sub-micrometer PM fraction, despite its potential to exert higher toxicity than larger particles (Negri et al. 2015).

Researchers are therefore engaged in the development of innovative air monitoring technologies able to sample PM down the sub-micrometer scale, but also to collect sufficient quantities to allow the physicochemical characterisation of the particles (Liu et al. 2023). However, even if advanced monitoring devices are being developed, currently their high costs do not permit to implement a satisfactory and up-to-date spatial and temporal analysis of airborne PM (Gozzi et al. 2016; Lucci et al. 2023; Liu et al. 2023).

An alternative approach to assess air pollution is biomonitoring (Käffer et al. 2012; Sæbø et al. 2012; Popek et al. 2017; Sorrentino et al. 2021). The managed honey bee species *Apis mellifera* has long been considered an ideal bioindicator of environmental pollution, and recently, it has been proven to be also an ideal tool for collecting spatial and temporal data on airborne PM at fine scales (Papa et al. 2021a; Pellecchia et al. 2023). In the following paragraph, the use of the honey bee to monitor airborne PM is presented and discussed.

## The use of honey bees to monitor airborne PM pollutants

When a living organism or part of it is used to define the characteristics of the biosphere it is referred to as biomonitor or bioindicator (Markert et al. 2003). *Apis mellifera* and related products as honey, pollen and wax are frequently used as biomonitors of pollutants.

The morphological and behavioral characteristics of this insect species, its ubiquity and short life cycle, the high reproduction rate, and the ease of breeding make it an excellent biomonitoring instrument. Furthermore, during the wide-ranging foraging activity, forager bees interact with all environmental domains (air, water, soil, and vegetation), collecting simultaneously a wide range of pollutants, from pesticides to trace elements, from volatile organic pollutants, polychlorinated biphenyls, and dioxins to low-level radioactivity (Bromenshenk et al. 1985; Tonelli et al. 1990; Leita et al. 1996; Satta et al. 2012; Losfeld et al. 2014; Zhou et al. 2018; Smith et al. 2019). Also, the use of bee products such as honey or other edible products (pollen, wax, and royal jelly) allows the collection of evidence for environmental pollutants to enter the food chain and to expose humans and pollinators to pollutant ingestion. In addition, being a living organism and a model species for ecotoxicological studies, the bee can be used to assess the environmental safety of chemicals (Papa et al. 2022).

**Fig. 1:**
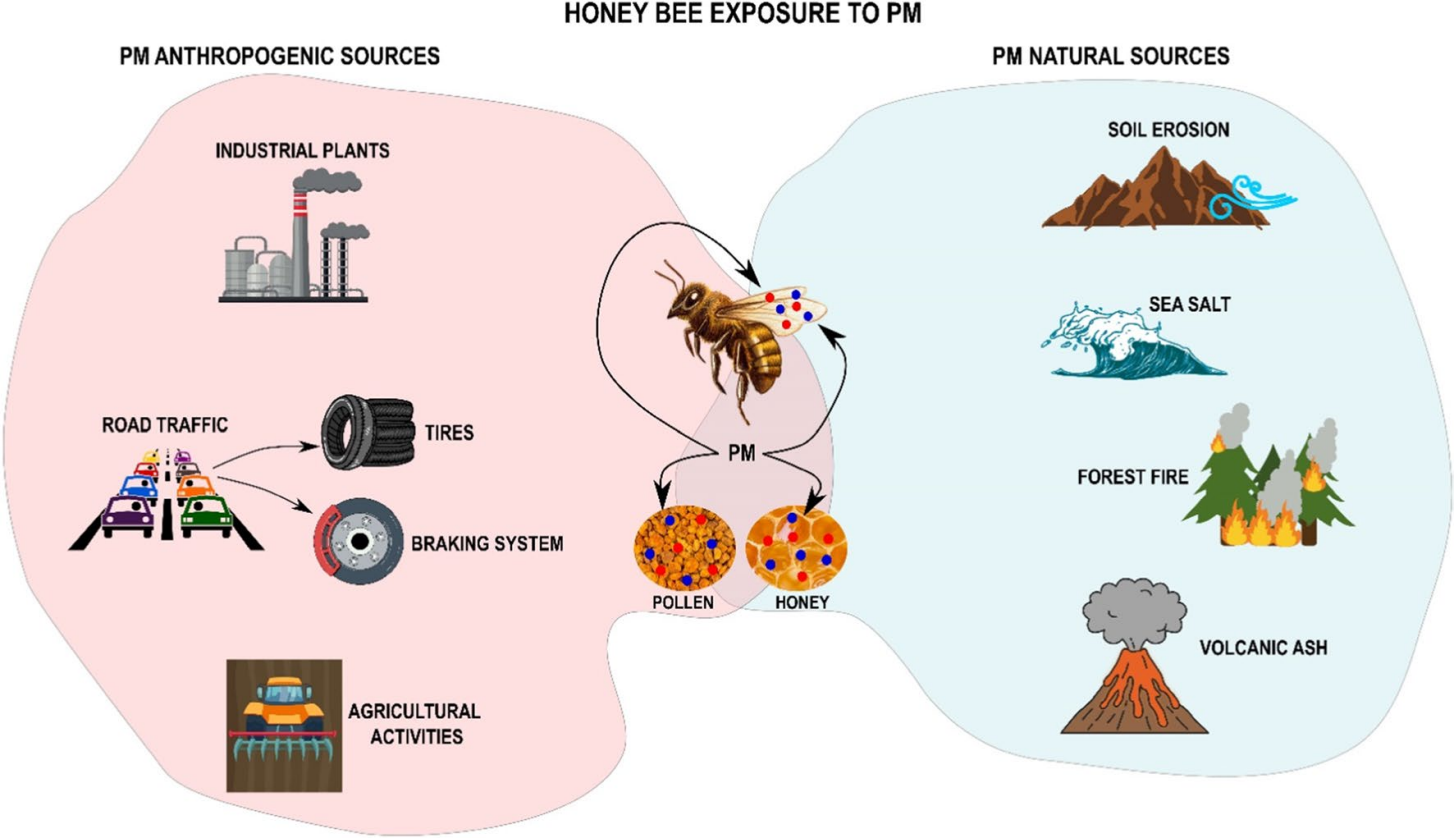
Overview of airborne PM exposure pathways for the honey bees. PM from both natural and anthropogenic origin adhere to the bee’s body and contaminate honey and pollen, exposing pollinators to ingestion

Since 2015 honey bees have been also used as bioindicators of airborne particulate matter (PM) (Negri et al. 2015; Pellecchia and Negri 2018; Papa et al. 2021a; Capitani et al. 2021). While foraging, worker bees accumulate fine dust on their bodies from both natural and anthropogenic sources which may also contaminate honey and bee pollen (Fig. 1) Papa et al. 2021a, b; Capitani et al. 2021).

The contamination of the bee’s body is due to the formation of electric charges during flight (Vaknin et al. 2000). Charge accumulation has been implicated in pollination: in bees, the friction surface is significantly increased by the presence of bristles and the accumulation of positive charges enhances pollen collection from flowers which are negatively charged (Vaknin et al. 2000). Bees and other pollinators also use the electric fields generated between their body and the flowers to choose nectariferous and pollen-bearing sources: already visited flowers seem less attractive because their electric charge is weakened (Vaknin et al. 2000; Khan et al. 2021; Liu et al. 2023).

However, the electric charges generated on the bees’ body also attract airborne inorganic particles. Particulate matter especially gathers on the anterior margin of the forewings of the bee (Negri et al. 2015). This may be due to the formation of the leading-edge vortex (LEV) (Negri et al. 2015), an aerodynamic mechanism generated during the insect flight which promotes the formation of a coherent vortical structure over the surface of the wings (Bomphrey et al. 2006, 2009). Such mechanism may be responsible of continuously trapping the airborne particles which are canalized by the air flow, eventually depositing on the wing edge where they adhere to the epicuticular wax and the hairs (Negri et al. 2015).

Airborne PM adhering to the bee can be characterised using a scanning electron microscope (SEM) coupled with energy dispersive X-ray spectroscopy (EDX) (Negri et al. 2015; Pellecchia and Negri 2018). SEM–EDX is a quick and non-destructive analytical technique, able to provide the size, morphology, and chemical composition of PM down to the ultrafine fraction. In the following paragraph, an overview of the methodology is provided, including limits and possible perspectives of this approach.

## Methods for the characterization of airborne PM sampled by honey bees 

The SEM method offers the possibility to measure particles on a distinct substrate, which in the case of honey bee impactors is represented by the insect’s forewings. Different imaging methods, as explained below, can be employed to bring particles out of the substrate background. Moreover, under specific image conditions, automatic particle analysis can provide all the relevant geometric parameters, such as particle maximum and minimum diameter, area, roundness, aspect ratio, etc. Particles below to nanometre in size can be imaged with a SEM and analyzed with EDX, and their morphology and chemistry connected with source apportionment. Indeed, mineral particles in most cases have a distinct habit, chemistry, and natural abundance, allowing the distinction from other particles or from same particles of different origin (i.e., anthropogenic).

The scanning electron microscope exploits an electron beam to probe objects. The resolution, and therefore the possibility to image small objects, is much higher as compared to an optical microscope that uses visible light: few nanometers against tenths of a micron, respectively, which allows the observation of the finest fraction of PM. Similarly, the depth of focus (i.e., the extent under which an object is in focus) is much higher, enhancing three-dimensional viewing and therefore the characterization of particle morphology. The most relevant advantage of SEM–EDX in comparison with all other techniques used for PM characterization is the possibility to determine individual particle chemistry on a sub-micrometre scale. 

### Scanning Electron Microscope (SEM)

A scanning electron microscope is constituted by an electron source (gun) either thermionic (tungsten hairpin or lanthanum hexaboride) or field emission (FEG) type. The latter gives a higher brilliance and coherency to the electron beam, therefore higher resolution. The electrons escaping from the filament are accelerated by the anode, placed just below, at the set accelerating voltage, usually between 2 and 30 kV, depending on the application. The optimal operational conditions are a complex combination of required resolution, nature of the sample (conducting, insulating, beam-sensitive, vacuum sensitive, etc.) and type of observation (morphology, composition, microstructure, etc.). In the study of inorganic airborne PM collected by bees, we found convenient to work at 15–20 kV.

**Fig. 2:**
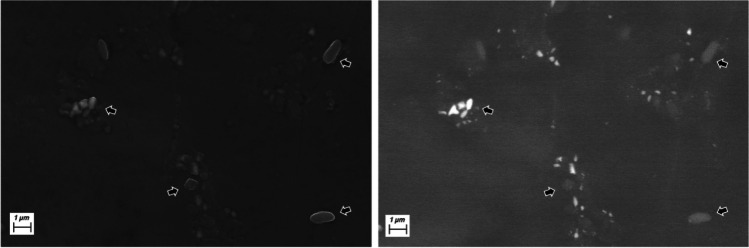
SE vs. BSE images. In the SE image (left) the fine contours of particles can be imaged (arrows). In BSE images, the same particles are less defined, but their different chemical nature can be appreciated: those with higher average atomic number (*Z*) appear brighter than those with lower *Z*

Each point on the sample hit by the beam produces a number of signals that are exploited to form images. The most commonly used signals to forming images are backscattered electrons (BSE) and secondary electrons (SE). The former are primary electrons of the beam that enter the sample and are diffused back by interaction with the sample atoms. The latter are electrons of the sample that receive energy from the incoming electron beam gaining energy as to escape the sample. BSE are high energy electrons (from the beam energy downward) which probe several hundreds of nanometres below the sample impact area of the beam and around it. They are sensitive to the average atomic number (*Z*) of the target (increasing with it) and can offer a resolution comparable with their penetration. SE electrons are low energy electrons (< 10 eV) able to escape only from the most superficial areas of the sample (2–5 nm), therefore suitable for high-resolution morphological images (Fig. 2).

Regarding particulate matter, both SE and BSE images are useful for its characterization as SE images give details of the morphology and topography of the particles down to the nanoscale, while BSE contrast gives information on differences in the atomic number (the higher the atomic number, the brighter the material appears in the image). The honey bee wings are made of low-*Z* compounds, such as chitin, proteins, and carbohydrates (Capitani et al. 2021). Therefore, high-*Z* particles clearly stand out such low-*Z* background. Often, particles of different origins such as different minerals, metals, and organic compounds can be easily distinguished on BSE images.

### Energy Dispersive X-Ray Spectroscopy (EDX)

**Fig. 3:**
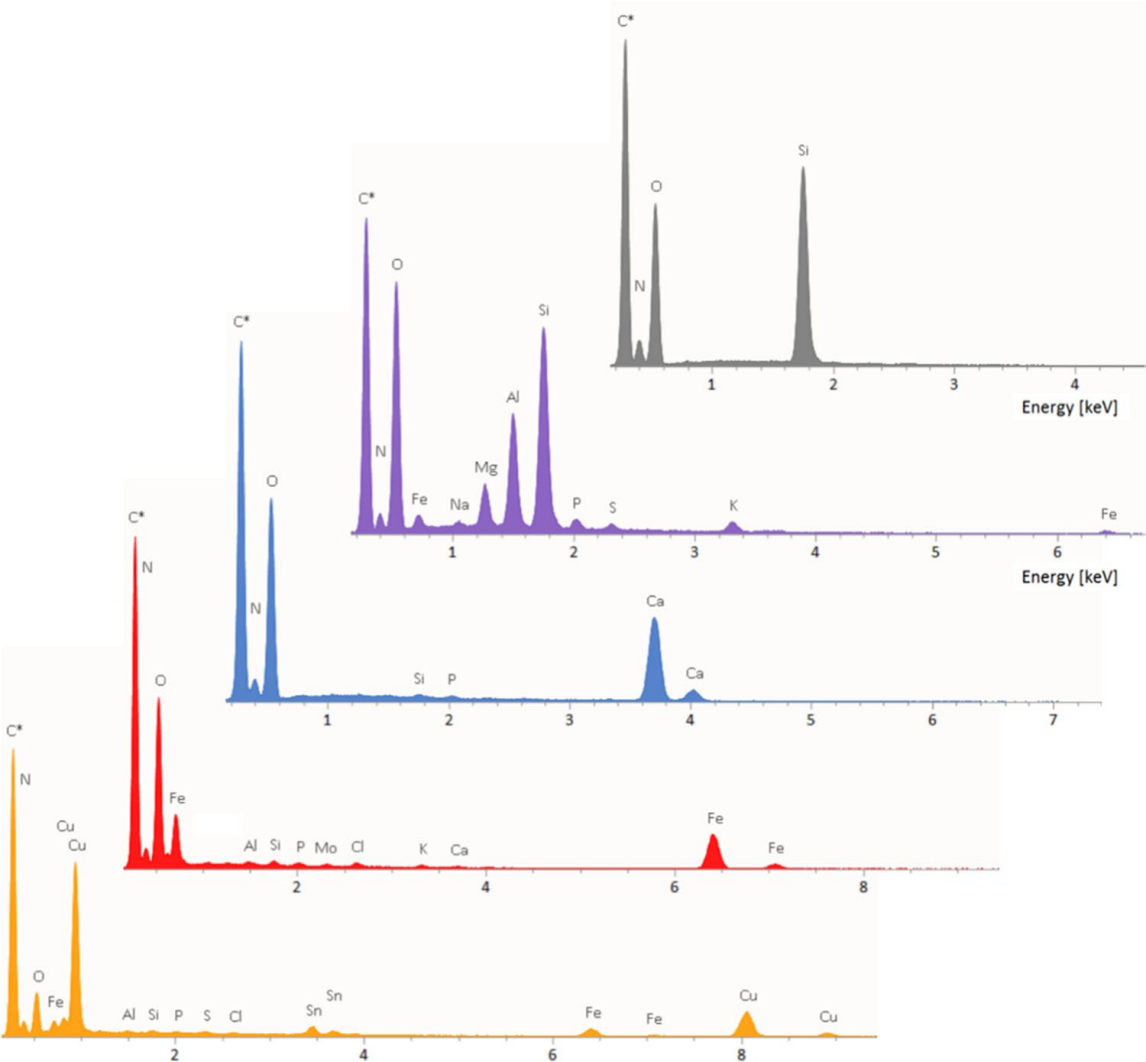
EDX spectra of PM particles. From top to bottom: quartz, clay mineral, calcite, iron oxide, and Cu-Fe metal particle. Minor peaks come from the wing and/or from other small particles attached to the main one

X-ray photons of energy characteristic of the impinged particle atoms are emitted whenever an electron of the beam releases its energy to an internal shell electron of the atom sample. The excited electron can therefore leave the sample, creating a vacancy. At the same time, an outer electron of the sample atom can fill the vacancy so created and an X-ray photon of energy equalling the difference between the outer and inner shells involved in the transition is emitted. The energy of the X-ray emission photon allows distinction among different elements present in the sample and their intensities allows relative proportions among different elements to be quantified. The ultimate result of the EDX analysis is a histogram, the EDX spectrum. Peaks in the spectrum, by virtue of their position in the energy bar, indicate the elements present in the sample, whereas their intensity is proportional to the raw abundance of that element (Fig. 3).

In order to transform intensity into element concentrations, some assumptions need to be made, as well as some corrections to the raw data (e.g., the ZAF method—Heinrich and Yakowitz 1975; Conconi et al. 2023). Briefly, the sample needs to be flat and the surface polished, and the interaction volume of the beam needs to be entirely confined within the sample. As we will see, these conditions are rarely achieved in PM analysis. However, the semi-quantitative EDX analysis can be sufficiently accurate for the purpose of particle identification.

### Example of particle identification: morphology and chemistry

**Fig. 4:**
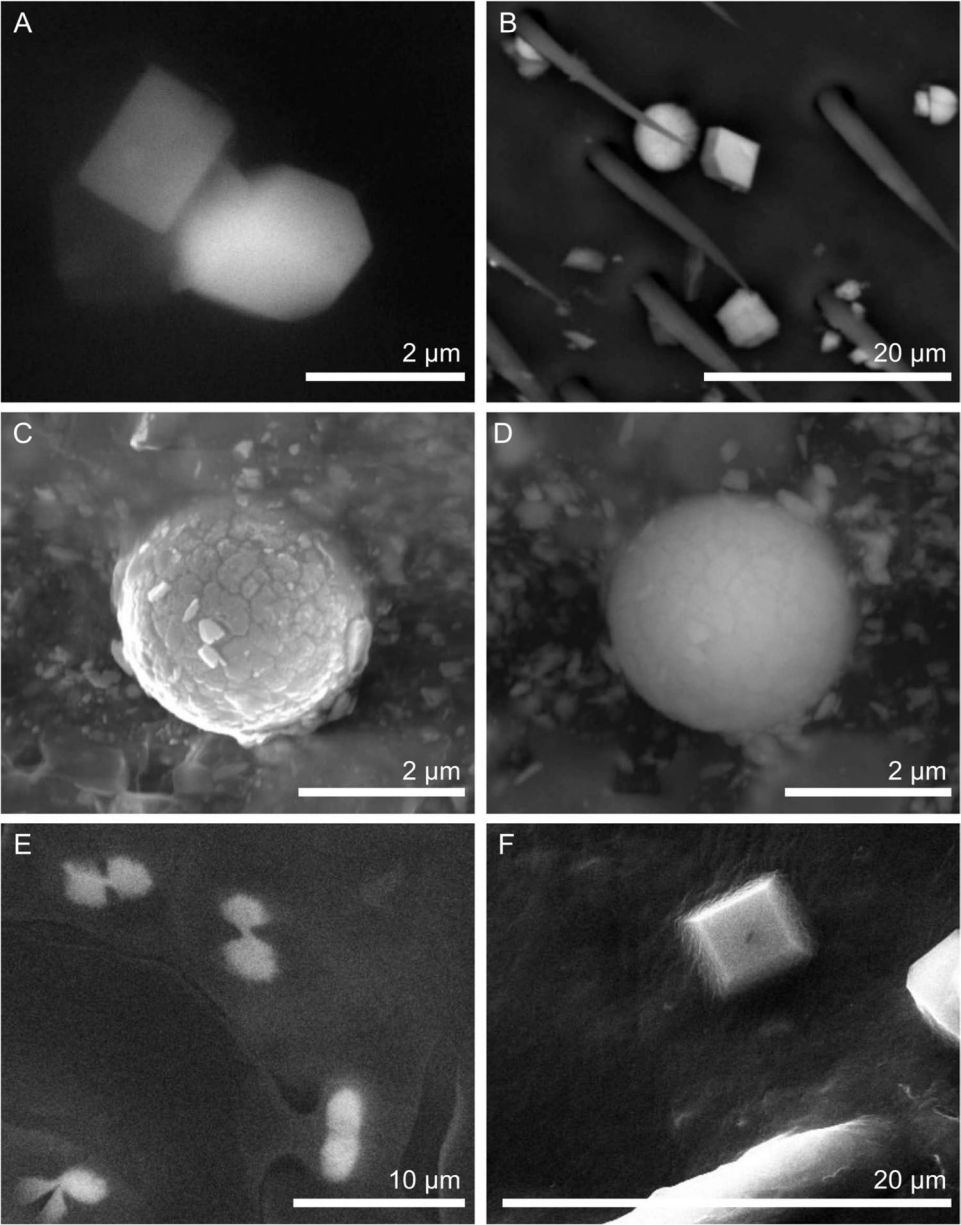
When the morphology comes to help (**A**) halite (Negri et al. 2015), (**B**) calcite (Plutino et al. 2022), (**C**, **D**) anthropogenic spherical particles (Negri et al. 2015), (**E**) crystals of calcium oxalate (Pellecchia and Negri 2018), and (**F**) a crystal of magnesium phosphate

Many crystalline phases have their own way to form a crystal habit and to part into smaller fragments if subjected to a mechanical stress, following geometrical constraints. Clay minerals, for instance, form platy crystals and part into lamellae (clay cleavage); sodium chloride forms cubes, as well the most common iron sulphide (pyrite, FeS_2_) and iron oxide (spinel, Fe_3_O_4_); calcium carbonate (calcite, CaCO_3_) forms rhombohedra and shows rhombohedral cleavage; asbestos minerals form fibres, etc. This is because the internal symmetry of crystalline materials is responsible of physical property anisotropy, which in turn affects crystal growth rate and mechanical strength variation with direction. On the other hand, anthropogenic compounds quenched from a high-temperature fume often show a spheroidal shape (Fig. 4).

However, things never go so simple in nature and sometimes the typical habit of a crystalline material may not form, as for instance when competing crystals growth closer in the same environment.

Regarding particle identification, the contribution of chemistry is a key step. Different phases commonly have different relative abundances of chemical elements. In this way, calcite can be easily distinguished from quartz for the presence of the Ca peak and the lack of the Si peak in the EDX spectrum, and vice versa (Fig. 3). Along the same, iron oxides can be distinguished from iron sulphides by the presence of the O peak and the lack of the S peak, and vice versa, and both can be distinguished from calcite and quartz because of similar arguments. Combining morphology and chemical signature, the nature of a particle in most cases can be assessed.

**Fig. 5:**
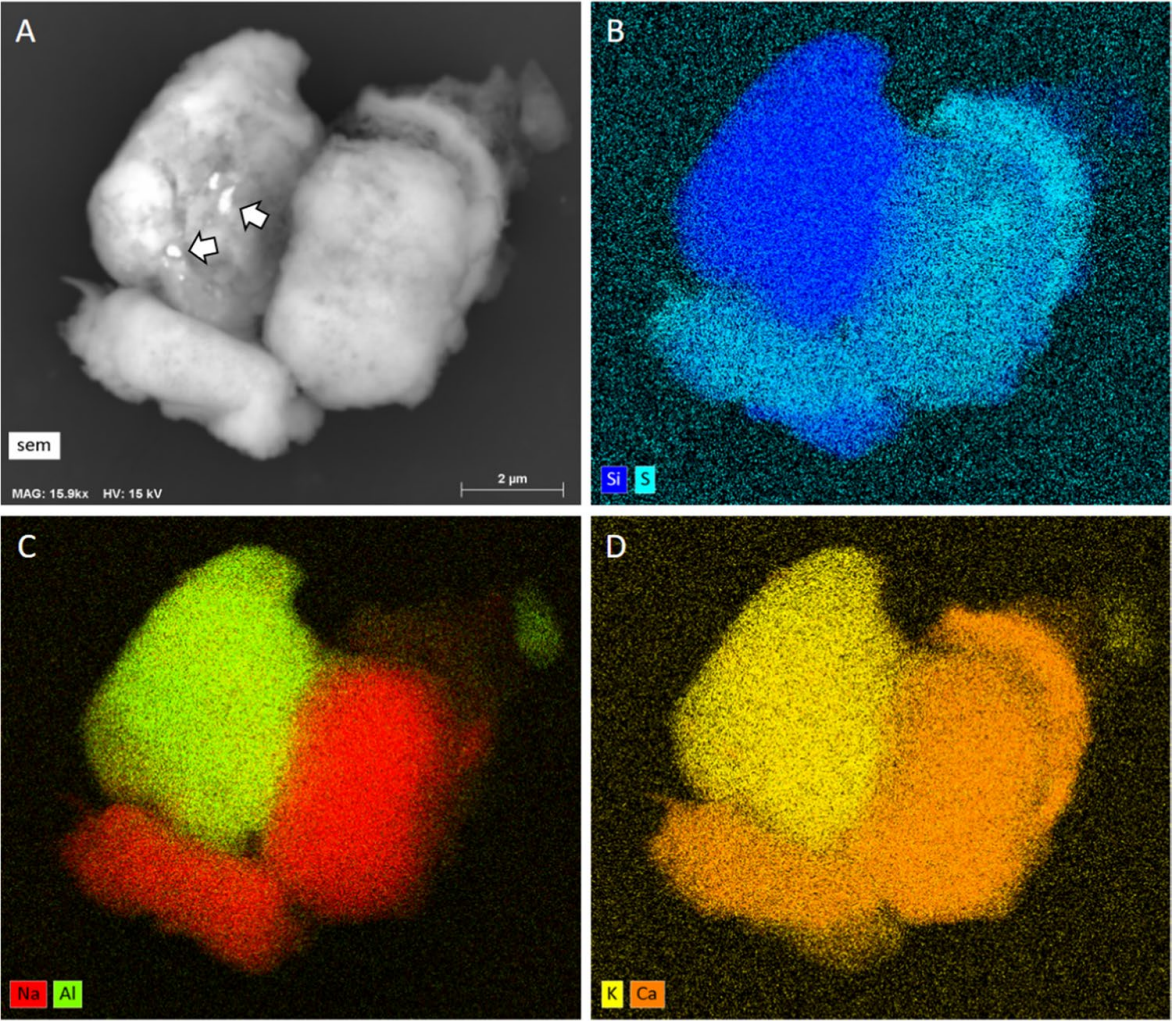
**A** BSE image and **B**–**D** elemental maps of a mineral aggregate. Combining these images, one can infer that the particle at the top left is Si-, K- and Al-rich, whereas the particles at the lower left and right are enriched in Na, Ca, and S, suggesting at least two different mineral phases. Moreover, the curve particle at the upper right is enriched in Ca and S, but not in Na, suggesting a third phase. Finally, the small, brighter particles arrowed in the BSE image in **A** suggest a forth, denser phase, that the Fe-map (not shown) revealed to be Fe-rich (after Capitani et al. 2021, modified)

In case of multiphase particles, different phases may be distinguished using elemental mapping and BSE imaging, as shown in the example of Fig. 5.

Finally, for a correct identification of the emission sources, SEM–EDX analysis must be associated to the geological characteristics of the area under investigation, along with a map of the possible anthropogenic emission sources of PM. This information is also essential for identifying human apportion of natural minerals, which occurs, for example, during construction activities or for activities that require natural raw materials. An example is the cement industry that uses limestone, marl, clay, and sand as natural raw materials. Minerals deriving from the grinding of rocks such as calcite, phyllosilicates, and quartz, can therefore end up in the atmosphere and be intercepted by bees (Pellecchia & Negri). However, the natural presence of those rocks in the area makes it almost impossible to distinguish between human and natural contribution from the erosion caused by atmospheric agents (Pellecchia and Negri 2018).

On the other hand, an anthropic contribution can be hypothesized when peculiar minerals are found in distinct geological settings: for example, the occurrence of barite (Ba sulphate), that is typical of hydrothermal deposits, in a valley of alluvial origin (Negri et al. 2015; Papa et al. 2021a; Capitani et al. 2021; Pellecchia et al. 2023); or the occurrence of halite, a natural sodium chloride also known as rock salt, in areas far from the sea and not including evaporite deposits, saline lakes or salt domes (Pellecchia and Negri 2018).

Table 1 lists the most recurrent inorganic dust particles detected so far in bees, their most probable natural or anthropogenic origin, some distinguishing morphological and chemical features detectable at the SEM, along with reference to published articles. Recent data on the two wasp species *Vespula vulgaris* and *V. germanica* used as mobile samplers of particulate matter in industrial areas are also included (Skaldina et al. 2023).

**Table 1:** Common inorganic dust particles detected in bees, origin, and distinguishing features

Particle	Main natural occurrences and potential dust sources	Main uses and possible anthropogenic sources	Distinguishing features in the SEM
Quartz^2,4^	Beach, river, and desert sands; soil and sediments; weathered sandstones, granitic rocks, and gneiss	Raw material for glass and abrasive industry; used as foundry sand and hydraulic fracturing proppant; present in construction and demolition wastes	Conchoidal fracture, irregular or prismatic habit; O and Si peaks
Feldspar^1,2,4^	Beach, river, and desert sands; soil and sediments; weathered granitic rocks, and gneiss	Raw material for glass and ceramic industry; used as fillers in paints, plastics, and rubber; present in construction and demolition wastes and dimension stone industry wastes	Orthogonal cleavage fragments; O, Si, Al, K ± Na peaks
Plagioclase^1,2,4^	Beach, river, and desert sands; soil and sediments; weathered gabbro, gneiss and amphibolite rocks	Raw material for glass and ceramic industry; used as fillers in paints, plastics, and rubber; present in construction and demolition waste and dimension stone industry waste	Orthogonal cleavage fragments; O, Si, Al, Ca, and Na peaks
Calcite^1,2,4^	Calcareous sediments and soils; weathered limestones and marbles	Raw material for the cement industry; used as soil conditioner; present in construction and demolition waste and dimension stone industry waste	Rhombohedral or scalenohedral habit; C, O, and Ca peaks
Dolomite^1^	Calcareous sediments and soils; dolostone and dolomitic marbles	Raw material for the cement industry; used as aggregate in the construction industry, as soil conditioner, as dimension stone and as source of magnesia (MgO)	Rhombohedral crystals with curved faces; C, O, Ca, and Mg peaks
Gypsum^1,2^	Evaporitic deposits in association with halite, anhydrite, sulphur, calcite, and dolomite	Manufacture of wallboard, cement, plaster of Paris; used as soil conditioning, as hardening retarder in Portland cement, as ornamental stone (alabaster)	Platy or splintery morphology; O, Ca, and S peaks
Barite^1,5^	Concretions and vein fillings in limestone, dolostone and sandstone; hydrothermal veins associated with sulphide ore	Constituent of brake pads; used as drilling mud, high-density filler for paper, rubber, and plastics; postmining-derived mineral	Very bright BSE yield; O, Ca, and S peaks
Clay minerals^1−4^	Soils, fine grained sedimentary rocks; weathered granites, slates and phyllites	Raw material for ceramic, cement, and lightweight aggregates; used as filler in paper, paint, plastic, and rubber, as lubricant in drilling fluids, as sealant in engineering applications, as absorbents for chemicals, oil, and water	Platy or splintery morphology; O, Al, Si, K peaks ± Na, Mg ± Fe
Cement minerals^2^	It is not a natural material	Binders in concrete for construction of buildings, roads, dams, ports, and decorative applications; present in construction and demolition waste	Fibrous or polyhedral morphology; O, Si, Al, Ca peaks ± Fe, Mg, S
Fe oxides and hydroxides^1−5^	Oxidized sedimentary deposits; accessory minerals in many primary rocks; alteration product of iron bearing rocks	Major constituent of brake discs; may result from alteration of iron infrastructures and mechanical parts	Very bright BSE yield; O and Fe peaks
Fe alloys^3−5^	Rare in nature; Fe–Ni alloys are present in meteorites	Fragments of worn mechanical parts or infrastructures	Very bright BSE yield; O, Fe and other metal peaks
Metallic Fe^3−5^	Extremely rare in nature (only one deposit in Greenland). May be present in meteorites	Fragments of worn mechanical parts or infrastructures	Very bright BSE yield; Fe peak
Galena^1^	Hydrothermal deposits; weathering and erosion of these primary deposits	Postmining-derived mineral; ore minerals for lead and rarely for silver (“argentiferous” galena)	Very bright BSE yield; cubic or rectangular fragments; Pb and S peaks
Fe, Zn, S, Si, and Ca^5^	Not interpretable as a single natural mineral phase; however, Fe-Zn sulphide (sphalerite) is abundant in hydrothermal deposits and Si and Ca may come from contaminant gangue minerals	Fe-Zn–S-Si-Ca have been found in tire wear particles	Very bright BSE yield; Fe, Zn, S and minor Si and Ca peaks
Fe, Ni, Cu, and S^6^	Not interpretable as a single natural mineral phase; however, pentlandite (Fe–Ni sulphide) and chalcopyrite (Fe-Cu sulphide) are widespread in nature	Fe–Ni-Cu–S particles have been found near to Cu-Ni smelting plants	Very bright BSE yield; Fe, Ni, Cu an S peaks
Spherical PM^1,2,4^	Although may be present in nature as a result of geological and biological processes and as micrometeorites, its occurrence is mostly anthropogenic	May be emitted from incinerators, chimney stacks, and furnaces	Spherical morphology with smooth or crusted surface. Many elements spectrum

^1^Negri et al. 2015

^2^Pellecchia and Negri 2018

^3^Papa et al. 2021a

^4^Capitani et al. 2021

^5^Pellecchia et al. 2023

^6^Skaldina et al. 2023

## Limits, tricks, and perspectives of the methodology

### Limits (light elements)

A major limitation of the SEM–EDX technique used in this approach is that low atomic number compounds, hydrocarbons or secondary PM for instance, cannot be easily distinguished from the background (the honey bee body), as they show similar contrast and response to X-ray analysis. Indeed, C and O are always present in the EDX spectra since both are major constituents of the honey bee body surface (chitin, wax, proteins, etc.) (Capitani et al. 2021; Pellecchia et al. 2023).

### Interaction volume, size of the PM, and chemistry

Except for light elements, the SEM–EDX analysis generally offers a higher characterization completeness in comparison to commonly used PM monitoring techniques. Notwithstanding, a reliable identification of the mineral phase by means of its composition and the correct measurement of the relative abundances of elements requires a particle volume larger than 3 ×  3 ×  3 µm and a flat and polished surface perpendicular to the electron beam. However, this is hardly achievable while studying PM contaminating the body of bees. If the particle is small and irregular in shape, the condition of constant electron/interaction volume is not met, the ZAF correction cannot be properly applied and the conversion of intensities into concentrations yields inaccurate results, making their interpretation more complicated. In-depth knowledge of the area of investigation (e.g., geological settings and presence and localization of PM emission sources) will definitely help the interpretation.

### Particle quantification

Regarding quantification, the number of particles collected by the honey bees during their foraging activity can be determined by counting the particles and measuring their dimensions. The functioning relays on the possibility to separate the particle from the background on the basis of the grey level (segmentation). Even if some contrast adjustment and image processing are possible, a prerequisite of the technique is a high-quality digital image where the particles could not be confused by their grey tone with the background or any other non-particle object. Another prerequisite is that particles are not in contact; otherwise, a single, larger particle is counted instead of several smaller ones. If this occurs, image processing filters (erosion and dilatation) aiming at separating the particles can be used. If all these prerequisites can be fulfilled, the particle analysis can provide the total number of particles per area, the size distribution, and a variety of geometrical parameters, such as particle roundness, aspect ratio, and maximum and minimum diameter. If the average density of the particles can be estimated based on the chemical composition, the mass of the collected PM can be determined. A critical parameter in doing such analysis is the image magnification, since it affects the particle size distribution: the higher the magnification the higher the number of small particles detected. Therefore, for the sake of a reliable representation of the particle size distribution, a thoughtful selection of different magnifications for the same sample is required. However, while allowing for the characterization of PM size, morphology, and chemical composition, this approach is time-consuming and its quantification efficiency should rely on the development of an automated image processing system, which may also improve accuracy and standardization.

## Bee ecotoxicology

In the last decades, several studies have investigated the effects of PM in pollinators, and some have explored such effects using the toxicology and ecotoxicology approaches, especially targeting heavy metals PM (e.g., Zn, Ti, Pb, Cd, and Ag; Table 2) (Özkan et al. 2015; Dabour et al. 2019; AL Naggar et al. 2020; Papa et al. 2021c). While field-based studies correlating the health of pollinators with levels of pollutant PM are necessary to understand the actual effect of airborne PM on human and ecosystem health in real-world conditions, till now, the existing studies are scarce or do not provide convincing results (Negri et al. 2020).

**Table 2:** Ecotoxicological effects of the exposure of honey bees to particulate matter

PM	Concentration	Effects	Publication
Ag-TiO_2_	312.845 mg/L	Death after 96 h	Özkan et al. 2015
CdO	0.01 g/mL	- Cytoplasmic proteolysis - Swollen nuclei - Abnormal distribution of heterochromatin - Microvilli damage - Mitochondria damage	Dabour et al. 2019
		- Significant difference in abundance of transcripts of detoxification and antioxidative defense genes - Significant decrease of acetylcholinesterase activity	AL Naggar et al. 2020
PbO	0.65 mg/mL	- Cytoplasmic proteolysis - Lysis of smooth endoplasmic reticulum - Swollen nuclei - Abnormal distribution of heterochromatin - Microvilli damage - Mitochondria damage - Abnormal Golgi bodies - Swollen lysosomes	Dabour et al. 2019
		- Significant difference in abundance of transcripts of detoxification and antioxidative defense genes - Significant decrease of acetylcholinesterase activity	AL Naggar et al. 2020
TiO_2_	5.865 mg/L	Death after 96 h	Özkan et al. 2015
	100 ng/µL 10 ng/µL	Impact on the gut bacterial communities	Papa et al. 2021c
ZnO-TiO_2_	6.351 mg/L	Death after 96 h	Özkan et al. 2015

The first toxicological study on honey bee evaluated oral exposure to heavy metal nanoparticles such as titanium dioxide (TiO_2_), silver loaded into TiO_2_ (Ag-TiO_2_), and titanium dioxide and zinc oxide (ZnO-TiO_2_) (Özkan et al. 2015). In this research, the authors tested the toxic effect of different concentrations of TiO_2_, Ag-TiO_2_, and ZnO-TiO_2_ and provided the median lethal concentration (LC50) values for the nanoparticles. The LC50 was assessed for 96 h and the TiO_2_, Ag-TiO_2_, and ZnO-TiO_2_ LC50 concentrations were found to be 5.865 mg/L, 312.845 mg/L, and 6.351 mg/L, respectively. Other studies investigated the sublethal effects of cadmium oxide (CdO) and lead oxide (PbO) nanoparticles provided separately and combined (Dabour et al. 2019; AL Naggar et al. 2020). The concentrations used corresponded to the 20% of the median lethal dose (LD50) and were comparable to the concentrations found in contaminated environments, namely, 0.01 mg/mL and 0.65 mg/mL for CdO and for PbO, respectively (Dabour et al. 2019). The results demonstrated that chronic exposure to sublethal concentrations of PM caused histological and cellular anomalies to midgut epithelium (e.g., mitochondrial swelling and lysis, irregular distribution, or/and condensation of nuclear chromatin). Congruent results were found by AL Naggar and colleagues in 2020; they recorded sublethal effects from chronic exposure (after 9 days) to sublethal concentrations of either CdO (0.01 mg/mL) or PbO (0.65 mg/mL), separately or combined. The sublethal effects caused significant difference in the transcript abundance of detoxification and antioxidative defence genes (cytochrome P450 gene CYP4G11) and superoxide dismutase (SODH2), and a significant decrease of acetylcholinesterase (AChE) activity (AL Naggar et al. 2020). Another study investigated the ecotoxicological effects of titanium dioxide (TiO_2_), i.e., a widely used compound in various industries such as food and cosmetics as a filler and whitening agent, albeit classified as a possible human carcinogen (Group 2B) by the International Agency for Research on Cancer (IARC). In 2021, Papa and colleagues (Papa et al. 2021c) demonstrated the sublethal effects of TiO_2_ sub-micrometer particles on the bee gut microbiota following acute and chronic exposure. In acute exposure, the probiotic *Lactobacillus* *kimbladii* was found to be significantly affected. Conversely, in chronic exposure, the *L.* *kimbladii* did not show inhibition but other important probiotic species were inhibited as *L. apis* and *L. melliventris* (Papa et al. 2021c). Finally, new studies demonstrated the presence of PM emitted from industries and vehicular traffic in honey and bee pollen (Papa et al. 2021a, b), suggesting the potential risk of pollutant particles entering the food chain and exposing bees and other pollinators to their ingestion (Fig. 1).

## Conclusion

Honey bees are eusocial insects, mainly known for their role in pollination, a fundamental ecosystem service securing plant biodiversity and ultimately the health of our planet. During their flights and foraging activity, honey bees interact with the lithosphere, hydrosphere, atmosphere, and biosphere; therefore, bees have long been considered ideal bio-monitors of pollutants, among which airborne particulate matter (PM) represents a ubiquitous component. As bees can fly several hundred meters around a hive and visit up to 1000 flowers per day, they represent an exceptional environmental sampler of PM under real-world conditions. Also, they mostly fly at human height; thus, the PM deposited on their bodies is the same reaching humans.

The use of bees offers many advantages over other PM samplers as particles collected on the bee’s body are ready to be analysed without further processing (e.g., preparation and extraction of filter material), thus minimizing the risk of contamination and sample loss. Furthermore, the bee acts similarly to a size-selective sampling unit with a cut-off of 10 μm in size, making also the finer fraction readily available for subsequent analysis. Finally, purchase and maintenance costs of bees are very low, as beekeeping is an easy and low-cost activity able to provide a potentially unlimited supply of PM samplers in almost all environments (Papa et al. 2022).

PM collected by bees can be subject to a chemical-physical characterization by means of a SEM–EDX, which may provide detailed information on the chemical form in which the particles composing the mixture are present. This can promote specific ecotoxicological studies on single substances alone or in combination.

Finally, the implementation of bee-monitoring stations may also provide further benefits to humans and the ecosystems. The honey bees can indeed deliver high-quality food to humans, such as honey, and many substances used in the pharmaceutical and cosmetics industry such as wax, propolis, pollen, royal jelly, and bee venom. Furthermore, bees promote plant reproduction through pollination and can be used to enhance crop yield and quality. Ultimately, the bees can be considered a cornerstone of biodiversity, food security, and food safety, supporting the principles of the “One Health” approach.
